# Changes in hip joint contact stress during a gait cycle based on the individualized modeling method of “gait-musculoskeletal system-finite element”

**DOI:** 10.1186/s13018-022-03094-5

**Published:** 2022-05-14

**Authors:** Binglang Xiong, Peng Yang, Tianye Lin, Jingli Xu, Yong Xie, Yongliang Guo, Churong Liu, QIzhao Zhou, Qizhong Lai, Wei He, Qiushi Wei, Qingwen Zhang

**Affiliations:** 1 The First Clinical Medical College, Guangzhou University of Chinese Medicine, Guangzhou, 510405 Guangdong China; 2 The Lab of Orthopaedics of Chinese Medicine of Lingnan Medical Research Center, Guangzhou University of Chinese Medicine, Guangzhou, 510405 Guangdong China; 3 Department of Joint Orthopaedic, the First Affiliated Hospital, Guangzhou University of Chinese Medicine, Guangzhou, 510405 Guangdong China; 4 Guangzhou University, Guangzhou, 510006 Guangdong China; 5 Brain Hospital Affiliated to Jinan University, Guangzhou, 510510 Guangdong China; 6 The Third Affiliated Hospital, Guangzhou University of Chinese Medicine, Guangzhou, 510240 Guangdong China; 7 Present Address: Second People’s Hospital of Shenzhen, Shenzhen, 518000 Guangdong China

**Keywords:** Gait, Musculoskeletal system, Finite element analysis, Hip biomechanics

## Abstract

**Objective:**

To construct a comprehensive simulation method of “gait-musculoskeletal system (MS)-finite element (FE)” for analysis of hip joint dynamics characteristics and the changes in the contact stress in the hip throughout a gait cycle.

**Methods:**

Two healthy volunteers (male and female) were recruited. The 3D gait trajectories during normal walking and the CT images including the hip and femur of the volunteers were obtained. CT imaging data in the DICOM format were extracted for subjected 3D hip joint reconstruction. The reconstructed 3D model files were used to realize the subject-specific registration of the pelvis and thigh segment of general musculoskeletal model. The captured marker trajectory data were used to drive subject-specific musculoskeletal model to complete inverse dynamic analysis. Results of inverse dynamic analysis were exported and applied as boundary and load settings of the hip joint finite element in ABAQUS. Finally, the finite element analysis (FEA) was performed to analyze contact stress of hip joint during a gait cycle of left foot.

**Results:**

In the inverse dynamic analysis, the dynamic changes of the main hip-femoral muscle force with respect to each phase of a single gait cycle were plotted. The hip joint reaction force reached a maximum value of 2.9%BW (body weight) and appeared at the end of the terminal stance phase. Twin peaks appeared at the initial contact phase and the end of the terminal stance phase, respectively. FEA showed the temporal changes in contact stress in the acetabulum. In the visual stress cloud chart, the acetabular contact stress was mainly distributed in the dome of the acetabulum and in the anterolateral area at the top of the femoral head during a single gait cycle. The acetabular contact area was between 293.8 and 998.4 mm^2^, and the maximum contact area appear at the mid-stance phase or the loading response phase of gait. The maximum contact stress of the acetabulum reached 6.91 MPa for the model 1 and 6.92 MPa for the model 2 at the terminal stance phase.

**Conclusions:**

The “Gait-MS-FE” technology is integrated to construct a comprehensive simulation framework. Based on human gait trajectories and their CT images, individualized simulation modeling can be achieved. Subject-specific gait in combination with an inverse dynamic analysis of the MS provides pre-processing parameters for FE simulation for more accurate biomechanical analysis of hip joint.

**Graphical abstract:**

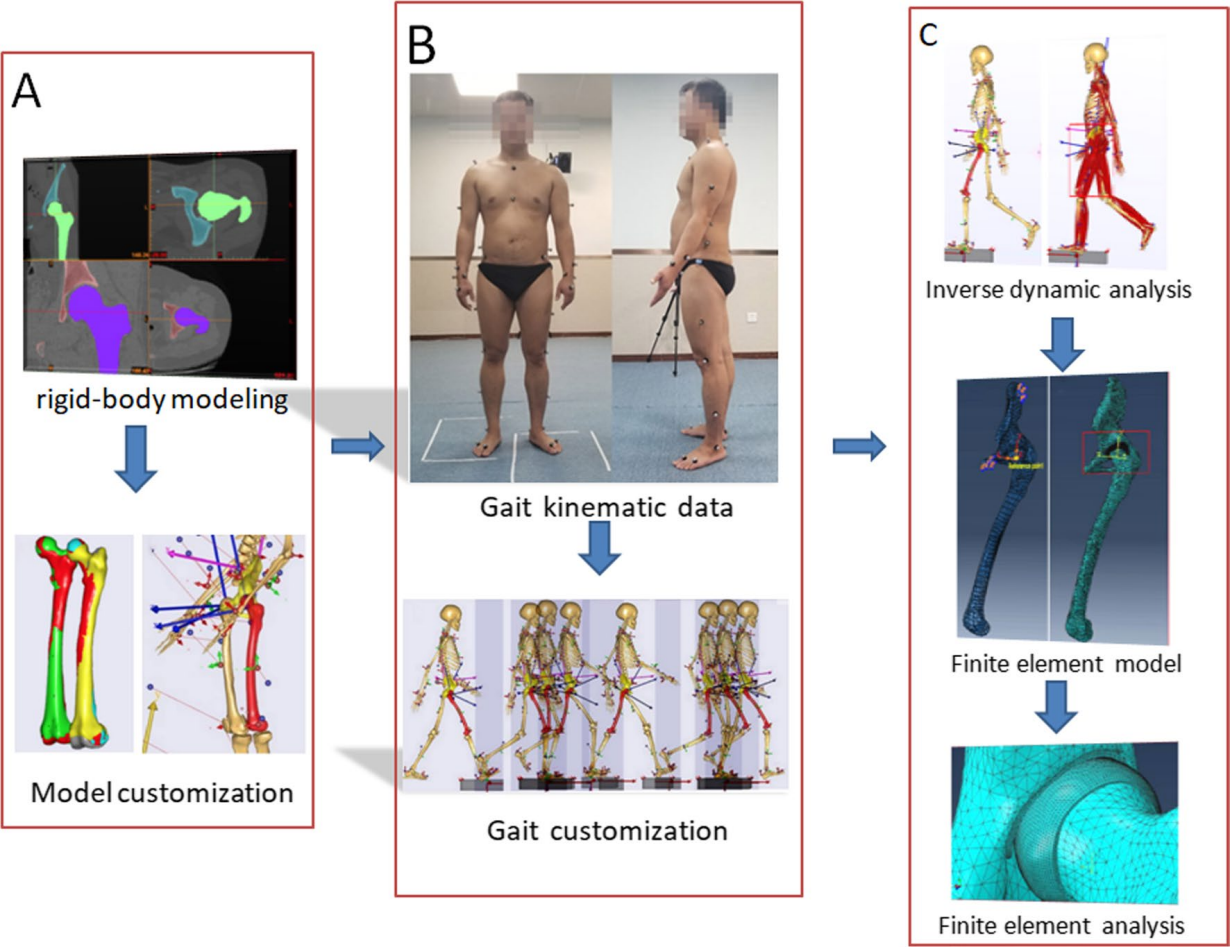

## Introduction

Hip arthritis as one of the most common hip diseases is projected to affect 411,000 people in the USA by 2030 [1]. Without appropriate treatment for long time, it will affect daily walking due to a chronic pain in the knee, the spine and other symptoms [2, 3]. Eventually, 572,000 patients could undergo the cost-consuming hip replacement surgery annually until the year 2030 [4, 5]. Therefore, studies about hip joint biomechanics to better understand the occurrence and development of hip diseases have significant clinical and economic implications. In order to simplify the calculation, many biomechanical studies on the hip joint only analyzed the bone model and only considered the effect of the joint force or gravity on the contact stress of the hip joint [6, 7]. To achieve more accurate hip biomechanic analysis, it is a high priority to consider integrated forces from skeletal structures, as well as surrounding muscles and ligaments of hip joint motion [8]. Moreover, in reality, the hip joint is always in constant motion to complete different activities. In a complete gait cycle, different mechanical states of the hip joint [9] remind us that gait characteristics should also be taken into account for biomechanics studies about the hip joint. However, most of the previous research literature of the finite element (FE) mechanics of the hip joint adopted a simplified model of one-foot standing, and it is believed that the hip bears the greatest force at this time [10–12]. We conducted this research with skepticism and established the “gait-musculoskeletal system (MS)-finite element” hip joint biomechanical research framework to explore the changes in hip joint contact stress during a gait cycle. Our research method overcomes the shortcomings of previous studies that did not consider muscle strength and gait data.

In previous biomechanical studies [13, 14], the muscle forces around one person’s joints obtained in the laboratory were loaded into another person’s FE model in the form of loads, while ignoring the differences in individualized muscle forces and the difference in hip joint morphology. These studies did not carry out individualized research. Some studies using the finite element analysis (FEA) have concluded that different pathological morphologies of the hip joint can affect the body-weight loading onto the articular cartilage [15–20]. The shape of the hip joint of standard-sized people is also slightly different. A study by Anderson et al. [21] reported that differences in hip morphology among individuals have a great effect on the contact stress distribution in the hip joint, as much as differences in the gait cycle. The FE model, the loaded muscle force and the gait data used in our study are from the same volunteer, which can truly result in a personalized study of hip joint biomechanics, eliminating the interference of other individual differences.

In this study, the human hip joint was individually simulated using the “gait-MS-FE” method to reconstruct a dynamic hip joint model more accurately. We assumed that the method used in our study was more reasonable than the previous and could achieve personalized biomechanics analysis. We provided a standardized modeling method for subsequent research on hip joint biomechanics. Our purpose was to analyze the changes in the contact stress of the hip joint during a gait cycle. The results obtained can be used to guide clinical decision-making, such as providing reference data for the acetabular rotation angle in hip dysplasia osteotomy. It can also be used for research on daily degeneration of hip joint prosthesis, which is beneficial to the improvement of prosthesis design.

## Methods

### Data collection

A healthy male volunteer (32 years of age, 70 kg, 171 cm) named model 1 and a healthy female volunteer (27 years of age, 51 kg, 162 cm) named model 2 were included in our study. The volunteer didn’t have any abnormalities in pelvic posture and hip morphology in pelvic X-ray and CT imaging. The following gait kinematic data of the two volunteers during the current experiment and the follow-up period were obtained. Our study has been approved by the Ethics Committee of the First Affiliated Hospital of Guangzhou University of Chinese Medicine (No. K [2019] 124).

#### Imaging data

The region from 1 cm above the highest point of the iliac crest to 3 cm below the trochanter was scanned using X-ray. The images of the pelvis and the femur included three anatomical views of cross section, coronal plane and sagittal plane were obtained by CT (0.5 mm slice thickness, 5 mm wavelength interval, image resolution of 1024 × 1024, 268 slices for male and 254 slices for female). CT images were saved as DICOM format and exported for 3D rigid-body modeling.

#### Gait kinematic data

**Fig. 1:**
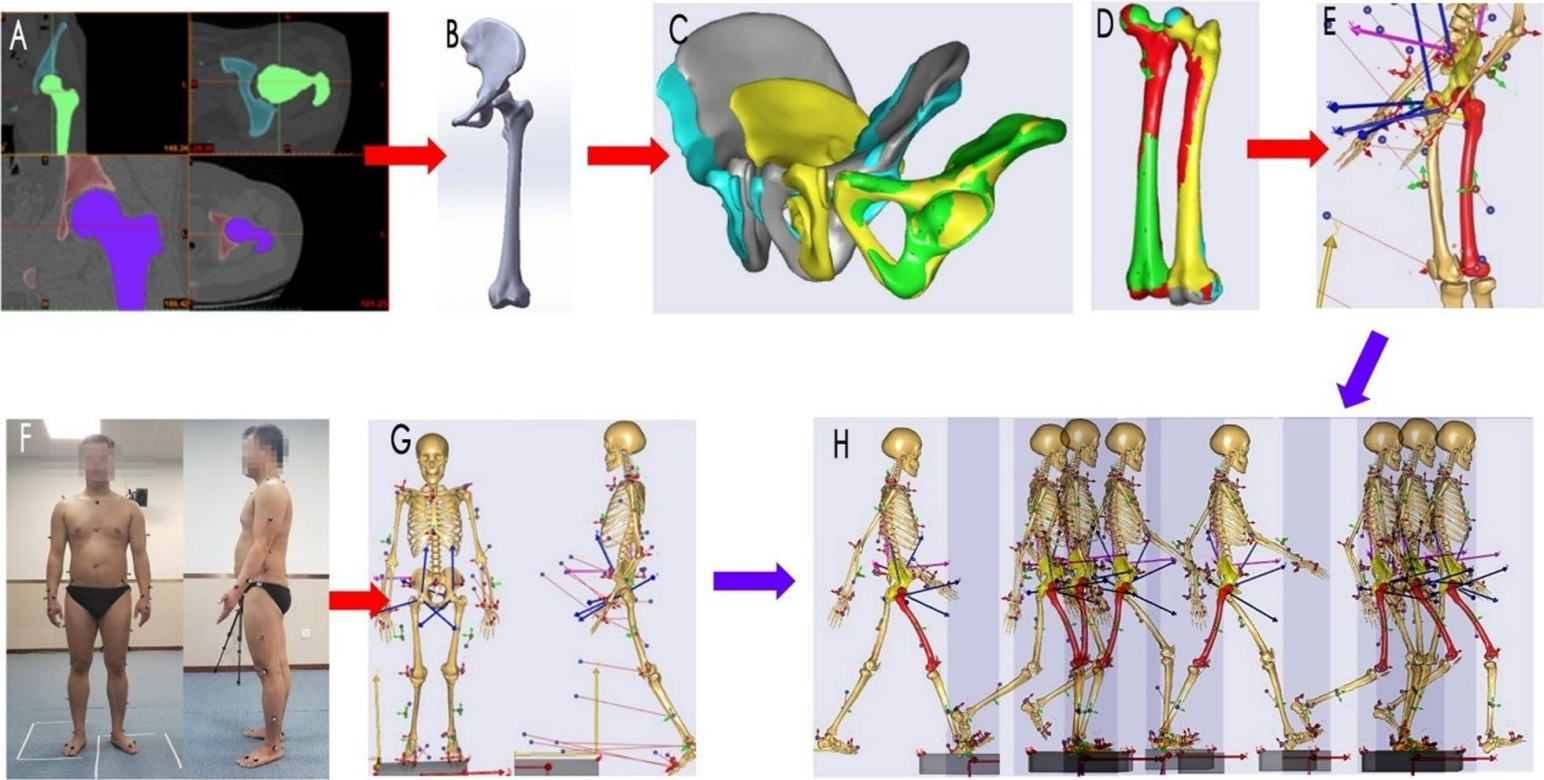
The workflow of a subject-specific musculoskeletal dynamic modeling. **A**, **B** Geometric reconstruction based on 3D CT data of normal hips; **C**, **D** the scaling of iliac and femur morphology; **E** a subject-specific musculoskeletal model after registration with geometric data; **F**, **G** collection and introduction of subject-specific gait data; **H** the dynamic hip motion of a subject-specific musculoskeletal model using kinematic analysis

Hip position data of standing posture and gait data of volunteers during walking were collected using the optical 3D gait analysis system (BTS Bioengineering, Italy). During the experiment, the two participants received adaptive training to walk continuously and freely prior to the tests. Then, they underwent 3 rounds of 5-m walk tests to ensure that both feet stepped into the force platform during at least one complete gait cycle in the one-way walking path. Infrared reflective markers were attached to the trunk, arms and legs. Afterwards, gait trajectories were formally collected and saved as the C3D file format for subsequent procedures (Fig. 1F, G).

### Subject-specific musculoskeletal dynamics simulation

The dynamic hip motion of musculoskeletal model was simulated in the AnyBody modeling system (AnyBody Technology Company, Denmark). Custom musculoskeletal dynamics and inverse dynamics were analyzed using a modified full-body model. As the function and application of AnyBody have been reported in similar studies [22], this article focused on the custom simulation process.

#### Model customization

To preliminarily adjust scaling of models, the body fat was measured using the built-in formulas in the AnyBody modeling system (volunteers’ BMI: model 1: 23.94/model 2: 19.4). To achieve a more accurate self-customization, DICOM data extracted from CT images were used for 3D reconstruction of the ilium and femur using Mimics software (version 16.0, Materialise, Belgium), (Fig. 1A, B). After reconstruction, 3D model files were imported into AnyBody for morphological scaling to determine the subject-specific registration of the pelvis and thigh segment of general musculoskeletal model in AnyBody. That model is based on spatial coordinates of the characteristic points of the iliofemoral models by point-to-point scaling codes. Moreover, the scaling was adjusted to match the starting and ending points of the muscles and ligaments surrounding the hip joint so that the anatomical features of human hip movements could be simulated more accurately (Fig. 1C, D, E).

#### Gait customization

**Fig. 2:**
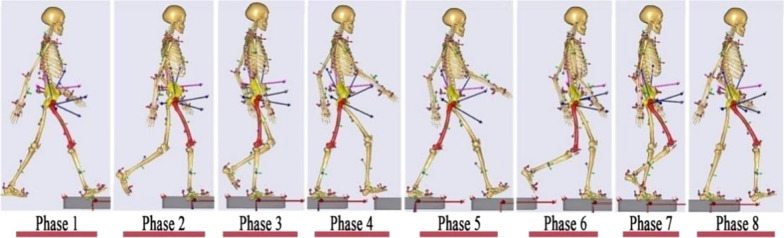
Schematic diagram of the 8 phases in a gait cycle using a subject-specific kinematic model. A gait cycle: phase 1, the initial contact phase; phase 2, the loading response phase; phase 3, the mid-stance phase; phase 4, the terminal stance phase; phase 5, the pre-swing phase; phase 6, the initial swing phase; phase 7, the mid-swing phase; and phase 8, the terminal swing phase

C3D files containing volunteers’ gait data were imported to AnyBody, and the locations of virtual markers alongside their 3D coordinates were adjusted to create the fully matched models in accordance with the locations of markers attached to the trunk, arms and legs of volunteers. The simulated models were optimized using parameters in the built-in kinematics optimization algorithm in AnyBody, which also simultaneously calculates the movement angles of the hip, ankle joints and the displacement of markers. The dynamic hip motion containing 8 phases in a gait cycle was simulated using the one of optimized multibody model (Fig. 2).

### Inverse dynamic analysis

**Fig. 3:**
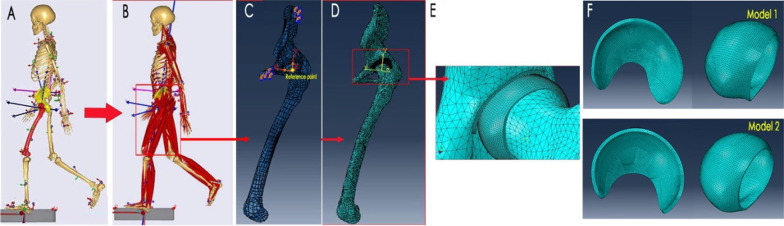
The musculoskeletal model and the FE model in the phase 2 of a gait cycle. **A**, **B** A subject-specific gait simulation using the kinematic analysis and inverse dynamic analysis; **C** the hip joint in an FE model (the yellow point is the center of the femoral head and also the reference point); **D**, **E** FE model (the yellow coordinate system has been registered and consistent with the femoral coordinate system in the AnyBody system); **F** cartilage model

Data obtained through kinematics optimization above were re-extracted for inverse dynamics analysis. Muscle importation was solved by formulating a third-order polynomial optimization problem. After the completed corresponding walking gait cycle of the musculoskeletal model during inverse dynamics loading was completed, the data of muscle forces and joint reaction forces of the legs were obtained (Fig. 3). Data of the muscle strength and the kinematics of the hip during a complete gait cycle were extracted for verification.

### FEA for the contact stress distribution

#### Geometric definition

Subject-specific geometric cartilage model is crucial for biomechanical analysis. Bone morphology has been reported to play an important role to predict cartilage stress [23], and it has also been shown that the optimal alignment of the joint was not sensitive to the choice of cartilage thickness distribution. Therefore, we performed a 3D dilation on the surface of the femoral head and lunate surface of acetabular fossa to reconstruct a constant thickness (1.8 mm) cartilage layers of the femur and acetabulum [23].

### Material properties and boundary conditions

As reported in study [24], the cartilage of a normal hip joint was modeled using homogeneous, isotropic and linear elastic materials, while the cortical bone and trabecular of the ilium and femur were modeled using homogeneous isotropic materials (Table 1).

**Table 1:** Properties of materials, the number of elements and elastic modulus in FE models

Components	Element type	Number of elements	Elastic modulus (MPa)	Poisson’s ratio
Cortical bone (femur + ilium)	C3D10	Model 1: 22827	15,100	0.3
		Model 2: 19881		
Trabecular bone (femur + ilium)	C3D10	Model 1: 45642	445	0.22
		Model 2: 41043		
Cartilage (femoral head)	C3D10M	Model 1: 56783	15	0.45
		Model 2: 51480		
Cartilage (acetabulum)	C3D10M	Model 1: 60100	15	0.45
		Model 2: 56104		

According to the method that has been reported in research [25], data of the 8 phases during a single gait cycle of the left foot were picked to analyze the contact stress of the hip joint during walking using the FEA. In the FE model, rigid transformation parameters of the hip during a gait cycle were adjusted using the kinematic data from the musculoskeletal simulation analysis and obtained by rotating coordinates of all unit nodes of the femur part. Assuming that the original coordinate of the femoral node was *P* (*x*_0_, *y*_0_, *z*_0_), the angles of hip rotation along the three axes of *x*, *y* and *z* were *θ*_*x*_, *θ*_*y*_, and *θz*, and then the three rotation matrices were calculated by Eqs. (1-1), (1-2) and (1-3):

1-1 $$A = \left[ {\begin{array}{*{20}c} 1 & 0 & 0 \\ 0 & {\cos \theta } & { - \sin \theta_{x} } \\ 0 & {\sin \theta } & {\cos \theta_{x} } \\ \end{array} } \right]$$

1-2 $$B = \left[ {\begin{array}{*{20}c} {\cos \theta_{y} } & 0 & { - \sin \theta_{y} } \\ 0 & 1 & 0 \\ {\sin \theta_{y} } & 0 & {\cos \theta_{y} } \\ \end{array} } \right]$$

1-3 $$C = \left[ {\begin{array}{*{20}c} {\cos \theta_{Z} } & {\sin \theta_{Z} } & 0 \\ { - \sin \theta_{Z} } & {\cos \theta_{Z} } & 0 \\ 0 & 0 & 1 \\ \end{array} } \right]$$

Based on a standard boundary condition described by Phillips et al. [26], encastre was applied at the top of the ilium and pubic areas. The rotation center of the femoral head, obtained using the least-squares spherical fitting method, was selected as the reference node. Nodes on the femoral head surface were constrained by the reference node using a kinematic coupling. The resultant force was applied at the reference point, and the direction of the resultant force at each gait phase was consistent with the reaction force of the hip joint (including the corresponding muscle forces [22] of the hip joint derived from inverse dynamics analysis). The interaction between the femoral head and the acetabulum was simulated by face-to-face contact, and the contact was assumed to be frictionless as it was used in study [27]. The cortical and trabecular bones were set to bind contact.

The mesh sensitivity was performed on a cartilage component rather than the skeletal for contact stress analysis. Since we mainly focus on the contact stress of the hip joint, a finer mesh was used. The maximum contact stress of the femoral head and acetabular was selected for the convergence test (Table 2). Three different mesh sizes were tested on the cartilage models, and the suitability was assessed based on the results of the contact stress analysis (the mesh selection criteria were defined as the changes in contact pressure and area with the difference between the meshes within 1%). According to the results, 1 mm size is selected to divide the cartilage.

**Table 2:** Convergence test for FEA models

Parameter	Type 1	Type 2	Type 3
Mesh size (mm)	0.5	1	1.5
Number of meshes (femoral head and acetabular cartilage)	146,866	116,883	87,689
Maximum contact stress (MPa)	5.97	6	6.11
Stress variation rate (%)	0	0.5	1.8
Contact area (mm^2^)	780.8	787.6	783.1
Contact area variation rate (%)	0	0.8	0.5

## Results

### Muscle force patterns

**Fig. 4:**
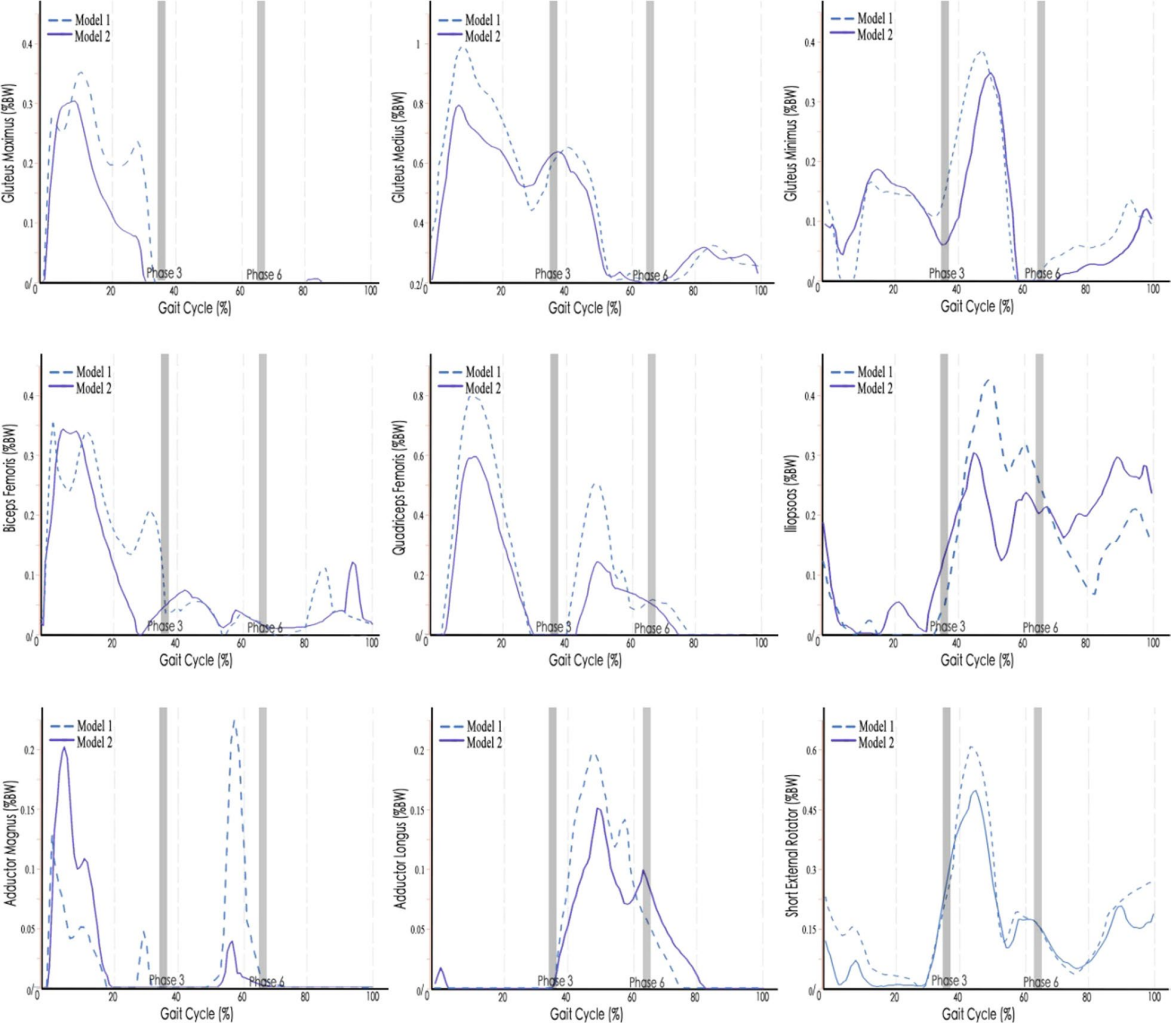
Muscle force patterns during a normal gait cycle using subject-specific musculoskeletal models

**Fig. 5:**
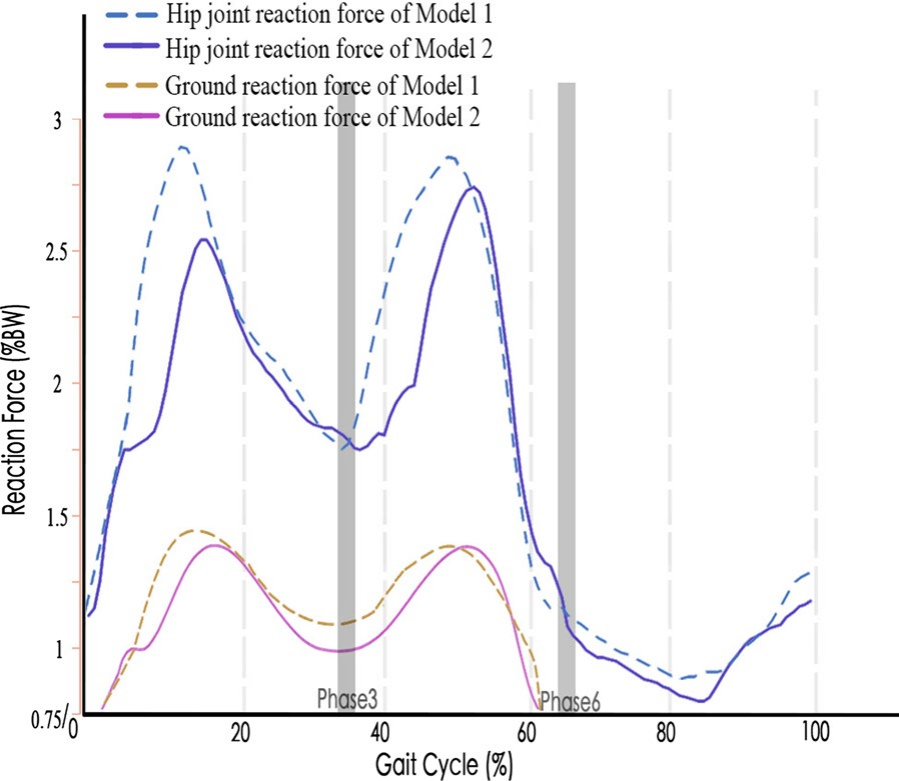
Hip joint reaction forces and ground reaction forces for subject-specific musculoskeletal models during gait cycle

After kinematics optimization, the AnyBody musculoskeletal models successfully completed gait trials containing complete gait cycles. Data of a single gait cycle (total time of a gait cycle: men: 1.17 s, women: 1.31 s) of the left foot were used for the inverse dynamic analysis. The simulated muscle force patterns in each gait phase during a normal gait cycle were basically consistent with the predicted results which have been reported in similar study [28]. The gluteus maximus, gluteus medius, biceps femoris, quadriceps femoris and adductor magnus showed peak muscle forces at the initial contact phase, while the short external rotator muscles consisting of the gluteus minimus, iliopsoas and adductor longus showed peak muscle forces at the end of the terminal stance phase (Fig. 4). The hip joint reaction force reached a maximum value of 2.9%BM at the end of the terminal stance phase. Twin peaks appeared at the initial contact phase and the end of the terminal stance phase, respectively. The change trend of ground reaction forces was roughly the same as that of Hip joint reaction forces (Fig. 5).

### Contact mechanics of the hip joint

**Fig. 6:**
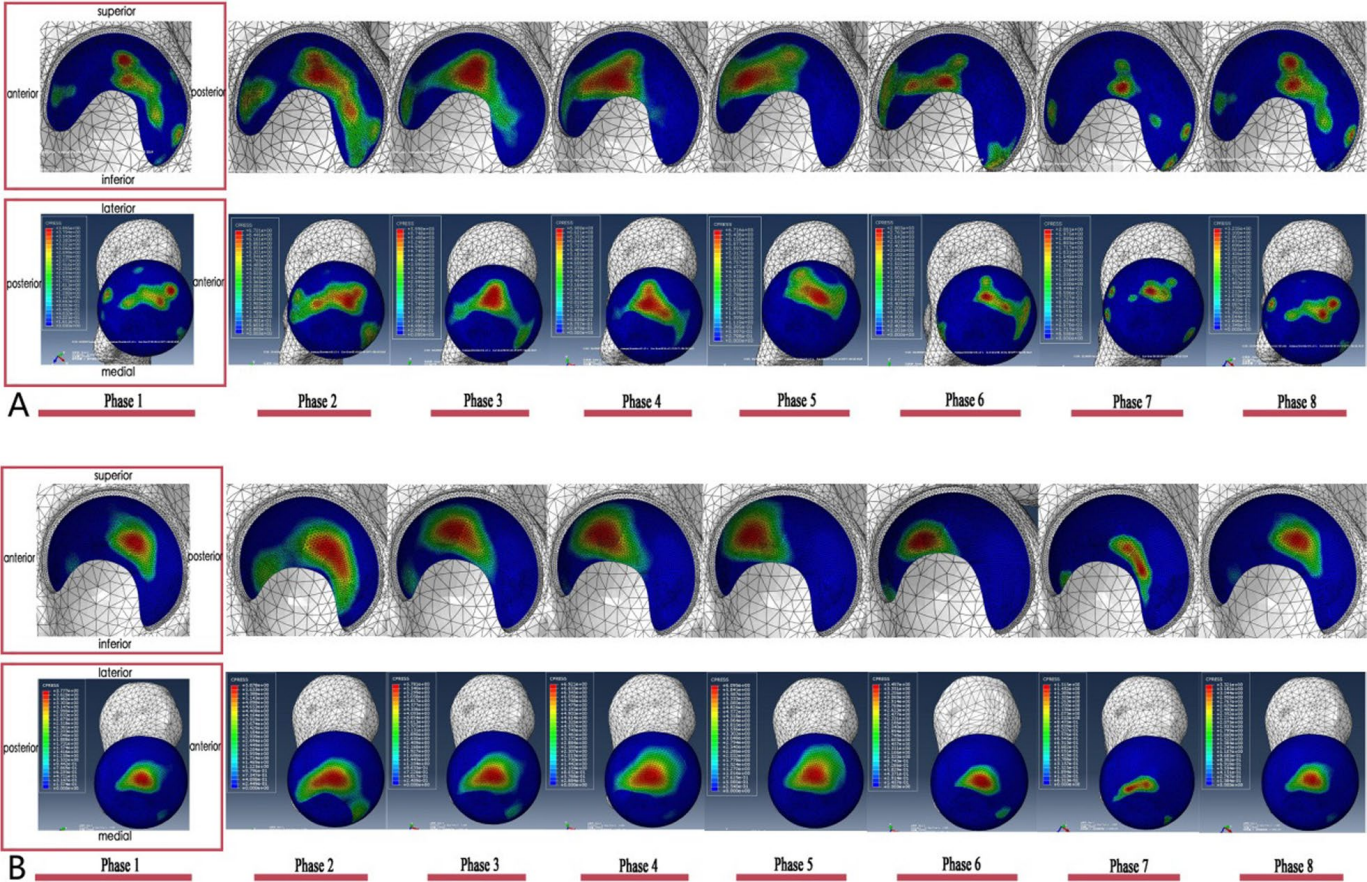
The nephograms of the contact pressure (CPRESS) on the acetabulum and femoral head cartilage surface during each phase of a gait cycle. **A** Model 1, **B** Model 2

The contact stress distribution of the hip joint during each phase of a normal gait cycle was analyzed by FEA. The results showed that the contact stress at each phase was consistent with the reaction forces of the hip joint. The FEA results showed the time-phase change characteristics of the contact stress distribution of the acetabulum. A peak contact pressure appeared at the loading response phase and the end of the terminal stance phase, during which the maximum contact stress reached 6.91 MPa in model 1 and 6.92 MPa in model 2 (Table 3). These results confirmed previous findings [29]. During a complete gait cycle, the contact pressure was mainly distributed at the top of the femoral head and the dome of the acetabulum, and moved from the anterior column to the posterior column of the acetabulum from phase 1 to phase 8 (Fig. 6). The contact areas were between 316.7 and 787.6 mm^2^ in model 1, 293.8 and 998.4 mm^2^ in model 2. Model 1 reached a maximum of 787.6mm^2^ at the mid-stance phase while model 2 reached a peak of 998.4 mm^2^ at the loading response phase.

**Table 3:** Hip joint reaction forces, peak contact pressure and contact area changes during different phases of a gait cycle

Phases of a gait cycle	Components (*Fx*, *Fy*, *Fz*) of hip joint reaction forces (N)						Peak contact pressure (MPa)		Contact area (mm^2^)	
	Model 1			Model 2			Model 1	Model 2	Model 1	Model 2
	− *F x*	− *F y*	− *F z*	− *F x*	− *F y*	− *F* z				
1	89.32	746.57	171.35	29.23	737.69	182.3	3.87	3.78	607.2	548.3
2	517.5	1706.6	636.6	241.27	1472.22	300.8	6.72	5.88	720.4	998.4
3	151.03	1211.7	404.1	103.97	1053.17	222.22	6	5.78	787.6	717.4
4	− 6	1562.5	442.43	67	1372.2	377.38	6.91	6.92	758.1	943.2
5	− 34.15	1837.55	342.37	11.67	1622.1	178.21	6.72	6.1	721.1	912.2
6	172.64	932.92	237.38	231.22	1033.38	334.17	2.88	3.5	589.3	672.2
7	91.23	478.7	243.72	45.22	344.83	177.31	2.06	1.52	316.7	238.8
8	92.67	717.3	172.74	37.18	710.26	177.9	3.24	3.32	586.4	426.1

Force components (*Fx*, *Fy*, *Fz*) corresponded to the local coordinate system of the thigh in MS models, *F*x*:* medio-lateral, *F*y: vertical, *F*z: anterior–posterior. Peak contact pressure and contact area were detected using the FE analysis

## Discussion

In this study, volunteers’ gait data were collected, and afterwards a musculoskeletal model was created and a reverse dynamic analysis was performed. We used the results of the inverse dynamic analysis as the boundary and load settings of the FE model of the hip joint. FEA was used to analyze the contact stress of the hip joint at each phase in a complete gait cycle. This study confirmed that the hip joint reaction was constantly changing during a complete gait cycle. Moreover, the contact stresses were the highest at the terminal stance phase at both models. Double peaks occurred at the loading response phase and at the end of the terminal stance phase, but not in the mid-stance phase.

Moreover, most of previous studies about contact mechanics of legs using the FE model were based on a standing position [6, 30–32], which could simplify the analysis and could not objectively reflect dynamic stress distribution. Therefore, it was easy to underestimate the damages of the contact stresses, while the relevant results could not truly reflect the human body. Studies have shown that excessive stress on the hip joint was the main cause of hip osteoarthritis [33, 34], which also reminds us that patients with clinical hip osteoarthritis are more prone to worsening in these two periods. Contact stress and maximum shear stress could be used to predict the fissuring of acetabular cartilage, which is one of the early symptoms of hip osteoarthritis in the body [35, 36]. The cross-cartilage changes of contact stress are related to the formation of larger shear forces [37]. Our study confirmed that the intensity and distribution area of contact stress changed with gait, which may damage articular cartilage and cause hip osteoarthritis. Other findings of our study were that the acetabular contact stress was mainly distributed in the medial part of the dome of the acetabulum and the anterolateral part of the top of the femoral head, moving from the anterior column to the posterior column of the acetabulum during a gait cycle. Therefore, in the daily diagnosis and treatment of femoral head necrosis, we must focus on the anterolateral part of the top of the femoral head to predict an early-on collapse, as this area bears the most concentrated forces at the early stage. This result is also helpful for guiding the daily rehabilitation exercise of patients with femoral head necrosis and for providing valuable recommendations for the selection of treatment regimens.

Few studies have analyzed changes in the dynamic contact stress distribution during a gait cycle. Brown et al. [38] implanted a resistive sensor on the surface of the acetabular cartilage in vivo to monitor the surface mechanics. The results obtained by this method, though relatively reliable, are highly invasive, technically demanding and cost-consuming. Wang et al. [39] analyzed the stress distribution during a normal gait cycle using the FEA and concluded that in a complete gait acetabulum contact stress presented bimodal distribution while the peak appeared in the starting phase and the support phase, respectively, with the maximum stress ranging from 4.2 to 3.3 MPa. Moreover, the acetabulum contact area reached the maximum of 1470 mm^2^, in the initial phase. Anderson et al. [40] showed that the maximum contact stress of the acetabulum during a walking gait was 10.78 MPa. After the FE analysis of 10 healthy volunteers, Michael D. Harris et al. [41] believed that the acetabular contact stress reached its peak (7.52 ± 2.11 mpa) when walking on the heel, while the average contact area occupied 34% of the total acetabular area. A study by Wu et al. [42] reported that in a gait cycle the maximum contact pressure reached 7.48 MPa. Their results are inconsistent with other studies which can be explained by their failure to consider impacts from the surrounding soft tissues such as muscles and ligaments. Besides, there are still differences between simulated data and gait trajectories of subjects. Li's [43] study uses the same to use the MS and FEA as this study for coupling modeling. The results showed that the maximum contact stress of the hip joint is 6.5 MPa presented to the initial contact phase, which is close to the results of this study. However, the used gait data were taken from public databases, not from the same researcher, and therefore it failed to achieve personalized research. Our research uses the “gait-MS-FE” method to add muscle strength around the joints to the model, which also reduces the influence of gait differences between individuals on the results of the study, so the results of this study are more credible.

The innovation of our research lies the fact that the method of “gait-MS-FE” is applied to the study of hip joint mechanics, and to personally study the changes in the contact stress of the hip joint during a gait cycle. This method can also be applied to the research of the contact stress changes of the hip in special positions and the contact stress of certain hip diseases. Genda et al. [44] analyzed and compared the contact stress between 112 healthy individuals and 66 patients, and the results showed that the contact stress in the normal acetabulum can be evenly distributed on the surface of the joints. Moreover, the articular contact stress in patients with joint dysplasia relatively concentrated on the anterior lateral edges of the acetabulum. However, the study only studied the contact stress in a stationary state and failed to pay attention to the characteristics of the entire gait. Robert et al. [45] studied the changes of the acetabular contact stress in 12 patients who had undergone periacetabular rotational osteotomy for nearly 10 years, which had a clinical implication for the treatment of hip dysplasia. However, the dynamic analysis of the whole gait process was ignored and the effect of the surrounding muscle force on the results was not considered. If the “gait-MS-FE” method is applied to the research of these diseases, it could make the research results more credible.

However, limitations in this study must be acknowledged. Like most studies about the FEA [46–49], all bone models were assigned with homogenization, while the actual bone density of different parts is uneven. The cartilage thickness of models was universal. In these regards, the reliability of our results may be reduced. Moreover, this study failed to consider the muscle forces on other joints such as the knee joint and the other leg, which may also have an impact on the contact stress of the hip joint. The influence of labrum in the contact stress of the hip joint has also been neglected. We utilized fixed boundary conditions at the top of the ilium and pubic area, while the fixed model is not representative of the in vivo environment. In the follow-up research, we will build the model more finely. In addition, this study failed to consider the influence of different walking speeds on the contact stress of the hip joint, Hu et al. [50] found that changes in walking speed may lead to changes in joint mechanics. We could explore the influence of different walking speeds on the contact stress of the hip joint in future researches. In this study, the result of maximum stress of 2 people with different BMI was too similar. So it cannot be ruled out whether BMI has an impact on the experimental results. In the future, a larger sample of research could be carried out.

## Conclusions

To sum up, the subject-specific gait in combination with an inverse dynamic analysis of the MS provides pre-processing parameters for FE simulation for biomechanical analysis of hip joint. In this study, the contact stress of the hip joint in a gait cycle was studied by constructing the “gait-MS-FE” method.

## Data Availability

Not applicable.

## Abbreviations

MS: Musculoskeletal system
FE: Finite element
BW: Body weight
FEA: Finite element analysis
